# Molecular Profiling, Characterization and Antimicrobial Efficacy of Silver Nanoparticles Synthesized from *Calvatia gigantea* and *Mycena leaiana* Against Multidrug-Resistant Pathogens

**DOI:** 10.3390/molecules28176291

**Published:** 2023-08-28

**Authors:** Sayab Khan, Muhammad Fiaz, Iqbal Ahmad Alvi, Muhammad Ikram, Humaira Yasmin, Junaid Ahmad, Amin Ullah, Zeeshan Niaz, Shubana Hayat, Ajaz Ahmad, Prashant Kaushik, Arshad Farid

**Affiliations:** 1 Department of Microbiology, Hazara University, Mansehra 21300, Pakistan; ksayab23@gmail.com (S.K.); iqbalalvi@hu.edu.pk (I.A.A.); shubanahayat@gmail.com (S.H.); 2 Department of Botany, Hazara University, Mansehra 21300, Pakistan; muhammadfiazhu@gmail.com; 3 Jamil Ur Rehman Center for Genome Research, HEJ, ICCBS University of Karachi, Karachi City 75270, Pakistan; ikramkalyar@gmail.com; 4 Department of Infectious Disease, Faculty of Medicine, South Kensington Campus, Imperial College, London SW7 2BX, UK; humaira.yasmin@comsats.edu.pk; 5 Department of Biosciences, COMSATS University, Islamabad 45550, Pakistan; 6 Department of Microbiology, Immunology, Infectious Diseases, Transplantation and Related Diseases, University of Rome Tor Vergata, 00133 Rome, Italy; ofridai116@gmail.com; 7 Department of Health and Biological Sciences, Abasyn University, Peshawar 25000, Pakistan; aminbiotech7@gmail.com; 8 Department of Clinical Pharmacy, College of Pharmacy, King Saud University, Riyadh 11451, Saudi Arabia; ajukash@gmail.com; 9 Independent Researcher, 46022 Valencia, Spain; prashantumri@gmail.com; 10 Gomal Center of Biochemistry and Biotechnology, Gomal University, Dera Ismail Khan 29050, Pakistan

**Keywords:** *Pseudomonas aeruginosa*, *Klebsiella pneumonia*, *Proteus mirabilis*, mushroom

## Abstract

The use of natural products isolated from mushrooms against infection, cancer diseases and other oxidative-stress-related diseases is one of the cornerstones of modern medicine. Therefore, we tried to establish a combination of medicinal mushrooms and nanotechnology possibly with the field of medicine for the development of antibacterial agents against these MDR strains. The aim of the research was to understand the molecular identification, characterization and antibacterial action of *Calvatia gigantea* and *Mycena leaiana*. The identification of fruiting body species via morpho-anatomical and molecular methods was necessary to analyze the genetic variability and phylogenetic relationships of mushrooms. Phylogenetic analysis revealed that *Calvatia* from Hunza, Pakistan, exhibited 98% resemblance to the previously discovered *Langermannia gigantean* (DQ112623) and *L. gigantean* (LN714562) from northern Europe, and *Mycena* (Pakistan) showed a 97% similarity to *M. leaiana* (MF686520) and *M. leaiana* (MW448623) from the USA. UV-vis, scanning electron microscopy (SEM), Fourier transform infrared spectroscopy (FTIR), and X-ray diffraction (XRD) were used for AgNPs’ characterization. The UV-vis absorption peak of 500–600 nm indicates the AgNPs’ presence. XRD results determined *Calvatia gigantea* AgNPs were nanocrystals and *Mycena leaiana* seems to be amorphous. In addition, SEM results showed the cubic morphology of *C. gigantea* with a diameter of 65 nm, and the FTIR spectra of fruiting body revealed the presence of functional groups—carboxyl, nitro, and hydroxyl—in AgNPs, which catalyzed the reduction of Ag+ to Ag0. Further antibacterial activity of mushrooms against MDR strains was determined via agar well diffusion assay, and Minimum Inhibitory Concentration (MIC) was estimated by qualitative experimentation using the broth dilution method. All experiments were conducted in triplicate. The results showed that the mushroom AgNPs, along with their synergy and nano-composites (with the exception of Ethyl-acetate), were shown to have zones of inhibition from 4 mm to 29 mm against multidrug-resistant pathogens such as *Acinetobacter baumannii*, *Staphylococcus aureus*, *Pseudomonas aeruginosa*, *Klebsiella pneumonia*, *Proteus mirabilis*, *Enterobacter cloacae* and *Escherichia coli*. The mushroom composites were active against most of the tested microorganisms whilst the lowest MIC value (10–40 mg/mL) was recorded against MDR strains. Hence, the present study suggested the possibility of employing compounds present in mushrooms for the development of new antibacterial agents, as well as efflux pump inhibitors.

## 1. Introduction

Higher Basidiomycetes, medicinal mushrooms and fungi have been part of the normal human diet for thousands of years, and in recent times, the amounts consumed have risen greatly, involving a large number of species [1]. Generally, edible mushrooms are utilized for nutritional and therapeutic purposes. The fruiting body cannot produce their food like chlorophyll-rich plants because they lack chlorophyll, indicating a short reproductive cycle [2]. Rural communities in the world utilize certain local wild mushrooms for food and medicine, but such indigenous knowledge is either not reported or poorly documented and unsystematically recorded [3]. Claims of their efficacy to be considered as food or medicine need to be scientifically verified prior to their acceptance. It is therefore necessary to know and correctly identify these mushroom species used by the locals as food or medicine [4]. The traditional techniques for identifying mushrooms include the morphological properties of the fruit body, selective inhibitors and culturing on different media to separate mycelia and indicator substrates. However, these techniques are complicated, time-consuming and may not accurately distinguish between closely related species. However, molecular identification has proven to be rapid, precise and sensitive for identifying mushrooms’ genetic similarities [5]. Molecular approaches used to identify mushrooms include matrix-assisted laser desorption/ionization time of flight (MALDI-TOF) mass spectrometry, rapid amplified polymorphic DNA (RAPD), internal transcribed spacer-restriction fragment length polymorphism (ITS-RFLP) and rDNA-ITS region sequence analysis [6]. Nuclear ribosomal DNA (rDNA), particularly the ITS section, is excellent for understanding mushroom genetics because ITS sections are highly conserved yet vary across species and are easy to amplify from modest amounts of DNA [7].

Mushrooms act as a valuable source for bioactive compounds used as medicine owing to their pharmacological availability, secondary substances and essential nutrients [8]. In addition to their vital function, mushrooms have antiviral, antiparasitic, anticoagulant, antiproliferative, antioxidant, anti-inflammatory, antibacterial, hepato-protective, antifungal, anti-diabetic, antitumor and insecticidal properties. With the development of bio-nanotechnology, the focus of researchers is now on AgNPs as a new potential antibacterial agent against MDR pathogens [9]. MDR can be defined as resistance to at least four classes of antibiotics used during the treatment of these infections. The emergence of MDR strains is often due to selective pressure from antimicrobial therapy. These drug-resistant pathogens are more pathogenic with a higher mortality rate than that of wild strains. To overcome microbial drug resistance, scientists are looking toward the development of alternative and novel drugs. Nanotechnology is expected to open some new aspects to fight and prevent diseases using atomic-scale tailoring of materials. Nanobiotechnology provides engineers the ability to develop nano-scale material characteristics with biological applications [10]. Depending on their size and form, AgNPs’ antibacterial activity alone or in combination emphasizes their importance in the war against MDR pathogens [9]. AgNPs are reported by scientist in numerous studies due to their prevalence in the environment and their capacity to adapt to harsh environments [11]. The size-specific antibacterial activity of NPs varies according to their synthesis, characterization and size, generally falling between 5 and 100 nm, where smaller NPs, as compared to large ones, exhibiting higher antimicrobial activity [12]. As NPs vary in shape and size, it is therefore possible to manage both of their characteristics via biological synthesis, which is more advantageous and of considerable interest. Many researchers have demonstrated the green synthesis of silver nanoparticles including bacteria, actinomycetes, fungi and plants. The green materials have been successfully used for silver nanoparticle synthesis, due to their potential medicinal properties, huge availability and faster rates of synthesis. *Calvatia* is an important medicinal mushroom belonging to the family *Lycoperdaceae*, and *Mycena* is a large genus, belonging to family *Mycenaceae*. It is a constituent of several remedies used for various treatments, such as wound dressing, diarrhea in calves and leucorrhea. Therefore, the combination of silver nanoparticles (AgNPs) and mushroom extract is rapidly developing antibacterial products that are biocompatible to overcome the obstacle of bacterial colonization and nano-engineering in the medicinal delivery of drugs [13]. The future prospects of mushroom crude extract and silver NPs (AgNPs) are addressed here, and there is a need to create environmentally acceptable approaches to synthesize tiny-size NPs that may be utilized commercially for beneficial applications. The objective of this study is to identify different mushroom species by utilizing the internal transcribed spacer-polymerase chain reaction (ITS-PCR) to explore the molecular variability of mushroom species, aid in mushroom taxonomy, as well as the characterization and evaluation of the antibacterial potential of synthesized mushroom extract and NPs against MDR pathogens.

## 2. Results and Discussion

### 2.1. Identification

#### 2.1.1. Morpho-Anatomical Study

**Figure 1:**
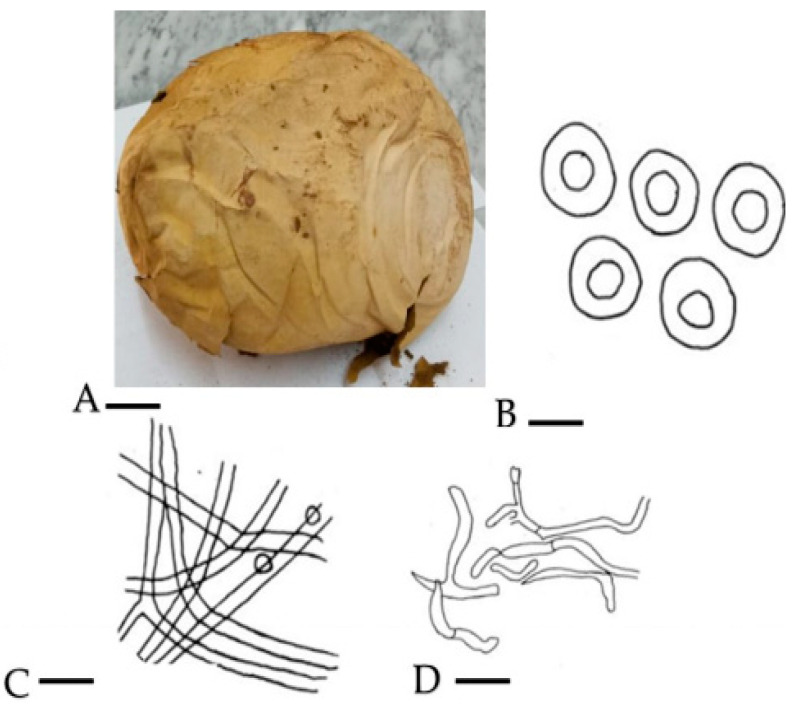
(**A**) Basidiocarp = 9 cm; (**B**) spores = 0.2 µm; (**C**) eucapillitial threads = 0.9 µm; (**D**) peridial threads = 0.7 µm.

Mushrooms have long been associated with humans and provide profound biological and economic benefits. Historical records have provided information on the consumption of wild mushrooms by humanity for their taste and pleasant flavor [14]. The morphology and anatomical parameters of *C. gigantea* revealed that basidiomata are globose to subglobose, soft white in the young stage, converting to brownish color with age, around 45 cm in height and 37 cm broad, an exterior epidermis attached with a substratum was not more than 1 mm thick and eroded in adulthood. The basidioma turns to brown spore dust with age. The appearance of the spots is sessile, globose to subglobose and brownish in color, and the diameter can be up to 3.5 to 4.2 m. The hyphae sometimes become yellowish in color, and the capillitia have a septate appearance, measuring around 2.4 m in width in KOH (Figure 1).

Materials Examined: Northern areas of Pakistan, Hunza, (2438 m) (7999 feet) from sea.

#### 2.1.2. Phylogenetic Analysis of *C. gigantea*

**Figure 2:**
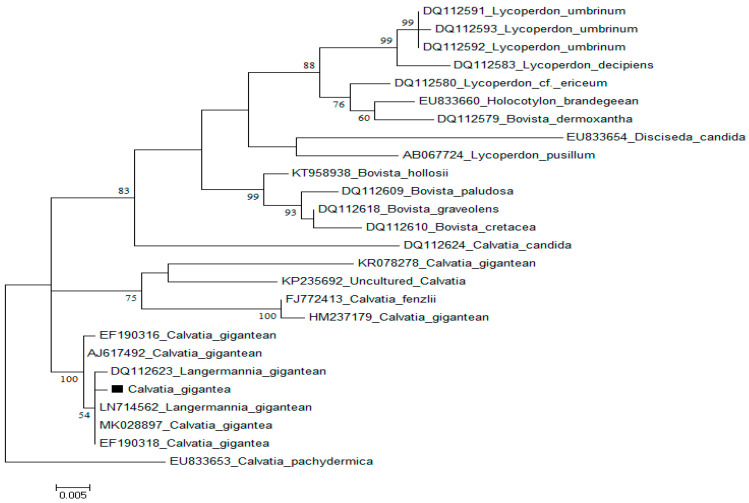
Using Maximum Likelihood technique with Tamura 3-parameter model to analyze *C. gigantea* ITS sequences. The final data set includes 30 nucleotide sequences and 293 positions.

The morphology and physiological parameters such as appearance, color, dimension, spores and form of the fungus on media, with other environmental growth preferences, is not sufficient for the identification of macrofungi. However, molecular-based tools offer better alternatives for the accurate identification [15] of wild medicinal and edible mushrooms, which will help in their proper documentation and effective exploration. ITS1F and ITS4 primers were used to amplify the nuclear rDNA ITS region of *C. gigantea*, and the consensus sequence (560 bp) in order to match with gene bank-submitted sequences was subjected to a BLAST search. BLAST analysis revealed that *C. gigantea* from Hunza, Pakistan, exhibited a 98% resemblance to the previously discovered *Langermannia gigantean* (DQ112623) and *L. gigantean* (LN714562) from northern Europe, with 100% query coverage and a 0.0 E-value for *C. gigantea* MK028897 from Poland (Figure 2).

For the phylogenetic analysis of *C. gigantean*, identical sequences of *C. pachydermica* (EU833653) were downloaded from Gene Bank [16]. The EMBL-EBI online MUSCLE program (http://www.ebi.ac.uk/) (accessed on 1 October 2021) was used to align downloaded gene sequences. The ITS data set includes approximately 1850 characters; the aligned sequences from the 5′ and 3′ ends of the ITS data set were removed, leaving just 500 characters in the final analysis. In total, 88 singletons, 190 prism-informative sites, 285 variable sites, and 277 conserved characters were included in the final alignment. The MEGA 6 tool with default settings was used to create the phylogenetic tree and reference sequences from GenBank.

### 2.2. M. leaiana

#### Morpho-Anatomical Study

**Figure 3:**
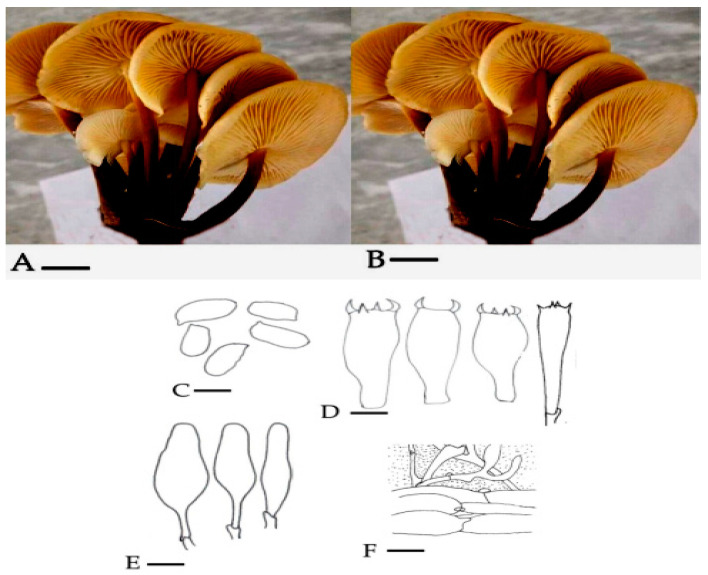
(**A**) = (pileus 1.5 μm); (**B**) = (lamellae & stipe) (1.5 μm); (**C**) = Ballistoconidia (10 μm); (**D**) = Basidium (5 μm); (**E**) = Cheilocystidium (12 μm); (**F**) = cuticle (8 μm).

*Mycena* was identified to genus level by means of observation of morpho-anatomical characteristics. It was the first attempt to describe the *M. leaiana* on both morpho-anatomical and molecular basis from Pakistan. The Pileus of *M. leaiana* was smooth, campanular, sticky, and shiny yellowish in color, around 1–2.5 cm in length with a striate border. Gills are adnate, yellow, and variable in length. The size of the central stipe is 3–6 × 0.15–0.3 cm, yellowish hollow and viscid in morphology. Basidia are clavate with 4 sterigmata, diameter of 27–38 × 6–8 m, and the spores are smooth, fleshy and elliptical about 7–10 × 4.5–5.5 μm in diameter. Its contain clamped parallel hyphae and top layer gelatinized with polymorphic cheilocystidia about 35–50 m and 10–13 m in size (Figure 3). Our study was supported by previous research of Das [17].

Materials Examined: khaki, district Mansehra KPK, Pakistan (873 m) (2864.17 feet) from sea.

### 2.3. Phylogenetic Analysis of M. leaiana

**Figure 4:**
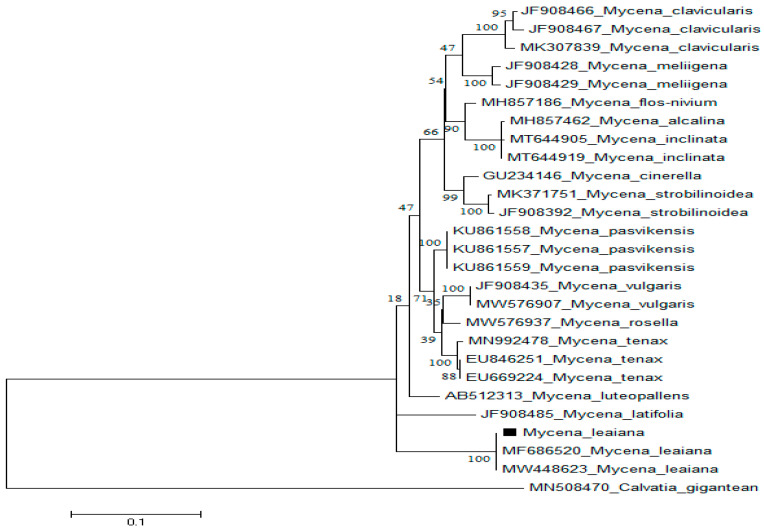
ITS sequences of *M. leaiana* were analyzed using the Tamura 3-parameter model with Maximum Likelihood technique. The final data set includes 30 nucleotide sequences and 290 variables.

ITS1F and ITS4 primers, was used to amplify the nuclear ribosomal ITS region of *M. leaiana*. Genebank sequences were used to BLAST consensus sequence (600 bp). With 100% 0.0 E-value as well as query coverage, *M. leaiana* (Pakistan) presented 97% resemblance to *M. leaiana* (MF686520) along with *M. leaiana* (MW448623) from the USA (Figure 4).

The identical sequences from GeneBank were used to construct the phylogenetic tree of *M. leaiana* (MN508470) [18]. All obtained sequences were aligned using the EMBL-EBI MUSCLE program (http://www.ebi.ac.uk/) (accessed on 1 October 2021). Once fully aligned sequences, 1800 nucleotides were removed from the 5′ and 3′ ends, only 540 sequences were utilized in the final section. The final alignment included variable sites (285), prism-informative sites (190), singletons (88), and 277 conserved characters. The phylogenetic tree was generated by using MEGA 6 tool with default setting.

### 2.4. Nanoparticle Characterization

#### 2.4.1. XRD Analysis

**Figure 5:**
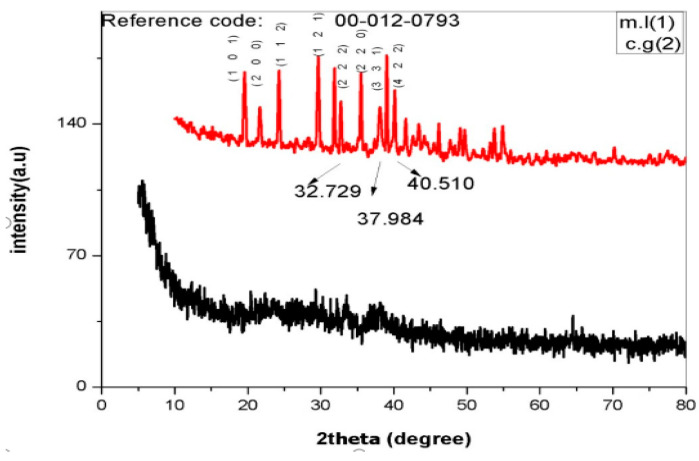
XRD analysis of AgNPs. m.l means *Mycena leaina* and c.g means *Calvatia gigante*.

XRD patterns of *C. gigantea* as well as *M. leaiana* showed a 2θ peak from 0 to 75 throughout the spectra. XRD pattern spectra clearly indicated the amorphous shape of *M. leaiana* and crystalline *C. gigantea* AgNPs structures. The particle size histogram of the silver nanoparticles obtained shows a broad distribution of particle size. The particle size ranged from 30 to 70 nm, and the average particle size comes out to be 65 nm. The XRD broadening peak was due to the moderate rate of reduction, indicating sizable NPs (Figure 5) [19,20]. *C. gigantea* XRD patterns showed seven peaks at 32.72°, 37.98° and 40.51°, corresponding to (220), (331) and (422) crystal planes [21]. XRD patterns were attributed to bio-organic crystallization due to some unknown peaks at 19.46°, 21.48°, 24.35° and 29.557° on AgNPs surface. The peaks were compared with an X-ray diffraction database. The same XRD patterns of *Azadirachta indica* NPs displayed characteristic peaks at 32.6°, 46.6°, 55.2°, 57.8°, 67.8°, 74.8°, 77.2° and 85.9°, which is in correspondence with (200), (220), (311), (222), (400), (331), (311) and (422) khl planes.

#### 2.4.2. UV-Vis Analysis

**Figure 6:**
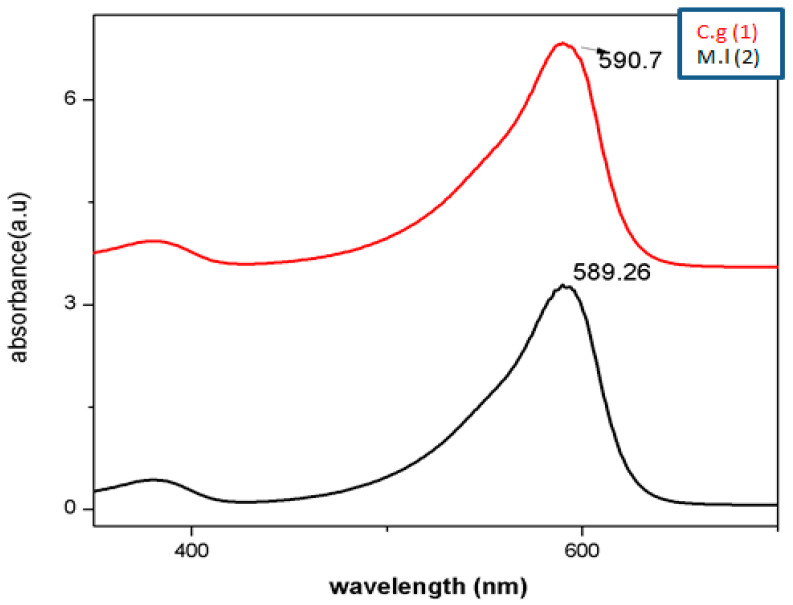
UV–vis analysis of AgNPs. M.l means *Mycena leaina* and c.g means *Calvatia gigante*.

Physiochemical and biological approaches have been employed to synthesize AgNPs in 1 h utilizing aqueous composites of *C. gigantea* and *M. leaiana*. However, when mushroom composites were incubated with distilled water (control), they remain their original color, whereas AgNO_3_-treated composites become brownish after 1 h due to SPR in the NPs. The solution of AgNO_3_ used alone as negative control showed no color change over 2 months. Our results coincide with a previous report [22]. The SPR peak of metallic NPs is highly dependent on their structure, as well as the chemical environment, which changes the position of the peak. The spectra exhibited SPR bands between 500 and 600 nm for both samples. The 600 nm peak is attributed to a tyrosine protein that stabilizes NPs or amide bands and tryptophan. The collective oscillation of the conducting electrons on the surface of the nano-sized particle absorbs visible electromagnetic waves, which confirms that silver particles are nano-sized [23] (Figure 6).

#### 2.4.3. SEM Analysis

**Figure 7:**
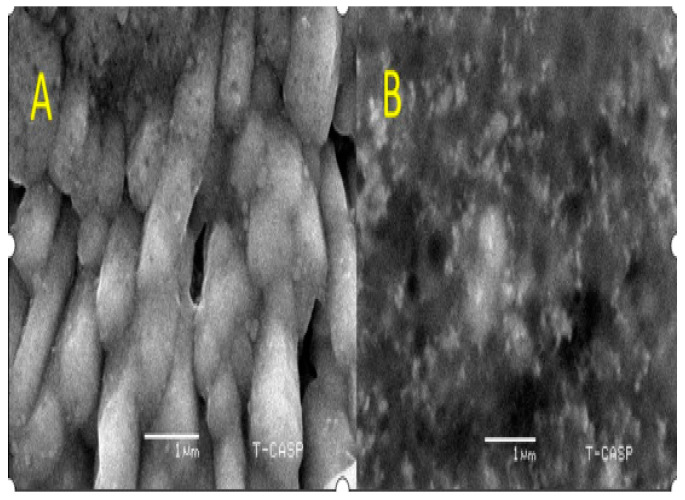
SEM analysis of *C. gigantea* and *M. leaiana. (***A**)—*Calvatia gigantia* SEM analysis ans (**B**)—*Mycena leaina* SEM analysis.

SEM further characterized the morphology of the AgNPs. *C. gigantea* AgNPs were 65 nm with a cubic structure, and *M. leaiana* was amorphous (Figure 7). According to the FTIR analysis, varying amounts of reducing and capping agents present in the mushroom extract yield diverse shapes and sizes of AgNPs. The shape of cubic-form silver NPs has been examined in previous research [24]. The extracts of *Boerhaavia diffusa* and *Dryopteris crassirhizoma* rhizomes operated as both reducing as well as capping agents in the green AgNPs. In less than 30 min, a cubic structure of *Boerhaavia diffusa* and *Dryopteris* NPs with a standard particle size of 5–65 nm was synthesized in the presence of sunlight/LED light [25].

#### 2.4.4. FT-IR Analysis

**Figure 8:**
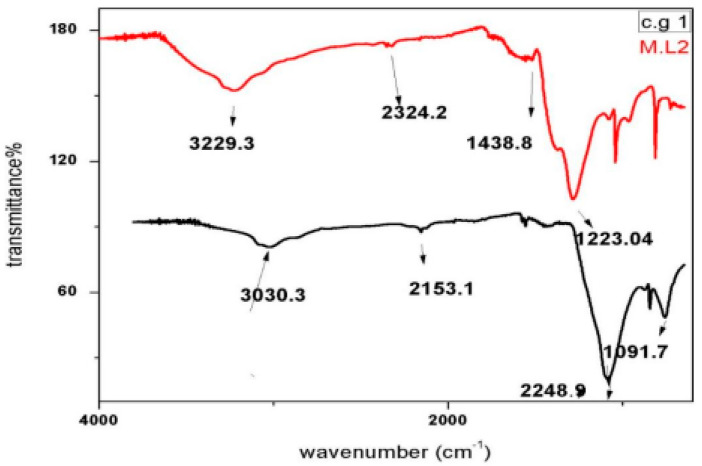
FTIR analysis of synthesized AgNPs from *C. gigantea.* M.L means *Mycena leaina* and c.g means *Calvatia gigante*.

Functional groups present in the nanoparticles of *C. gigantea* along with *M. leaiana* were identified using FT-IR spectroscopy, reducing Ag ions and the capping reagent that maintains bio-reduced AgNPs. The absorption bands of *C. gigantea* were centered at 1091, 2153, 2248 and 3030 cm^−1^ and those of *M. leaiana* at 1223, 1438, 2324 and 3229 cm^−1^. The appearance of crests that were prominent were located at 3030 and 3229 cm^−1^, suggesting hydroxyl stretching (O-H), which is linked with the movement of alcohol as well as phenolic groups from mushroom AEs to AgNPs. Another group of crests was observed at 2248.9 and 2324.2 cm^−1^, associated with nitro stretching (-NO_2_ group). The peak of *C. gigantea* at 1091 cm^−1^ was associated with carboxyl stretching (C-O) vibration, attributed to Ester linkages, while the peak at *M. leaiana* 1223 cm^−1^ was associated with acetyl stretching (CH_3_-CO groups). Therefore, from the FTIR results, it is clear that the synthesized nanoparticles were surrounded by proteins and amino acids, which may be responsible for the stability of the silver nanoparticles. Proteins and capping agents are essential for stabilizing the synthesized AgNPs, and O-H, C-O, N-O as well as acetyl groups can transform Ag ions to metallic Ag (Figure 8) [26].

### 2.5. Antibacterial Potential of Organic Solvent

Due to excessive antibiotic usage and inappropriate prescription, MDR infections represent a substantial threat to the therapeutic system worldwide [27]. In this study, mushroom species were tested for their antibacterial activities. Our screening results confirmed that mushrooms exhibited moderate to remarkable antibacterial activity against several bacteria, including resistant and multidrug-resistant strains. No antibacterial analysis of *C. gigantea* or *M. leaiana* has been conducted to date. Comparisons of our results have been made with the antibacterial activity of mushroom extracts belonging to different taxonomic groups.

#### 2.5.1. Methanol

**Figure 9:**
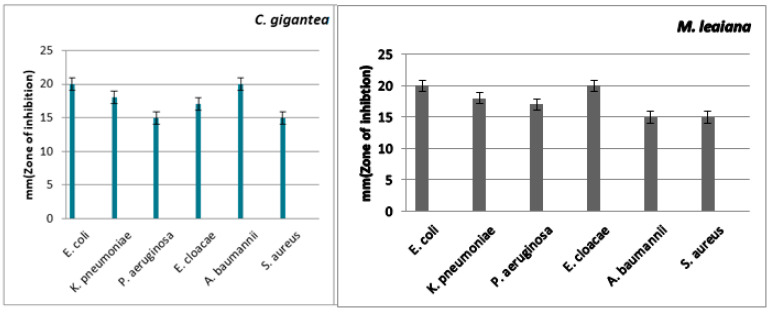
Antibacterial action of methanolic composite of *C. gigantea* and *M. leaiana* against MDR pathogens.

*C. gigantea* methanolic extract has strong anti-MDR pathogen activity. The ZOI against *K. pneumoniae* was 23 mm, and the minimum was 11 mm against *E. cloacae*. The largest ZOI of *M. leaiana* methanolic extract was 20 mm against *E. coli and E. cloacae* and displayed the minimum size against *A. baumannii* and *S. aureus*, which was 15 mm (see Table 1 and Table 2) (Figure 9). Previous research revealed that the methanolic composite of *G. lucidum* showed similar efficacy against *K. pneumoniae* (21 mm) and *E. coli* (20 mm) [28]. Methanolic extracts were shown to have the most common antibacterial actions against Gram-negative bacteria as compared to Gram-positive pathogens. Gram-negative bacteria contain an outer membrane that is hydrophilic because of the occurrence of lipopolysaccharide molecules that enable smaller lipophilic compounds to move via the membrane, which is necessary for any antibacterial solute to penetrate the microorganisms’ membrane. Methanol has low water solubility and can easily penetrate into the outermost membrane of Gram-negative pathogens, disrupting their metabolism and cellular function, leading to the death of the microbes [29].

#### 2.5.2. Ethyl Acetate Extract

The antibacterial effect of *C. gigantea* ethyl acetate showed significant efficacy against *A. baumannii* and *E. coli* among tested microorganisms. The maximum ZOI was displayed against *E. coli* (23 mm) and minimum against *A. baumannii* (16 mm). *M. Leaiana* ethyl acetate extract is more effective than *C. gigantea* against *E. coli, P. aeruginosa, K. pneumoniae and E. cloacae*. The highest ZOI measured against *E. coli* was 10 mm and the least was 4 mm against *K. pneumonia* (see Table 1 and Table 2). Nevertheless, no action was shown against *S. aureus* and *A. baumannii*. In previous research, ethyl acetate extracts of oyster mushrooms exhibited minimal and *reishi* showed no inhibitory zone against most of examined organisms [30]. Based on our results and earlier investigations, ethyl-acetate mushroom extract was ineffective against most MDR bacteria. This might be because the antimicrobial compounds include polar flavonoids and terpenoids that can be extracted using a less polar solvent.

#### 2.5.3. n-Hexane Extract

The n-hexane-derived mushroom extract exhibits strong antibacterial potential against examined MDR bacteria. The highest ZOI was examined against *P. aeruginosa* (22 mm), while the smallest was 16 mm against *S. aureus*. The largest ZOI of *M. leaiana* n-hexane extract was measured against *E. coli* (19 mm), while the smallest was against *K. pneumoniae* and was 10 mm (see Table 1 and Table 2). *C. gigantea* and *M. leaiana* n-hexane extracts were more effective against all tested pathogens than ethyl-acetate extracts. At 100 μL, the n-hexane extract of *V. sativa* showed the maximum antibacterial activity at 100 mg/mL concentration against *E. coli* (40 mm) [31].

#### 2.5.4. Aqueous Extract (AE)

*C. gigantea* AE is effective against all bacteria except for *E. cloacae*. The maximum ZOI was detected against *E. coli* (24 mm), and the minimum was 15 mm against *A. baumannii*. When compared to *C. gigantea*, the *M. leaiana* AE demonstrated a modest level of antibacterial effectiveness against MDR bacteria, but no impact was seen against *S. aureus*. The most significant ZOI against *E. cloacae* was 14 mm, and the least against *K. pneumoniae* was 5 mm. The MDR pathogens showed resistance against (AE) of *C. gigantea* and *M. leaiana* up to 50%. According to prior research, the most active constituent is water insoluble, therefore more active extracts were dissolved in low polar organic solvents. Due to the lipid composition of diverse microorganisms’ membranes and the permeability of mushroom components, the aqueous extract has less antibacterial action than the organic extracts. We can also see differences in the size of the ZOI among bacteria [32].

#### 2.5.5. Pure Water Extracts (PWE)

The pure water extract of mushroom samples showed good antibacterial activity against microbes. The maximum ZOIs were 20 mm against *S. aureus* and *K. pneumoniae* and the minimum was 17 mm against *E. cloacae. M. leaiana*’s peak ZOI was detected to be 22 mm against *S. aureus* and the lowest was 16 mm against *K. pneumonia* (see Table 1 and Table 2). Aqueous extracts from both species of mushrooms represented high activity against most of the sensitive and resistant clinical isolates. Aqueous extracts’ bacterial inhibition halos against Gram-positive bacteria varied between 8 and 25 mm for *B. edulis* and 8 and 18 mm for *N. luridiformis*. Aqueous extracts also demonstrated high bacterial inhibition halos against *P. aeruginosa,* with values ranging from 13 to 21 mm and 7 to 17 mm for *B. edulis* and *N. luridiformis*, respectively [33].

#### 2.5.6. AgNPs’ Antibacterial Activity

**Figure 10:**
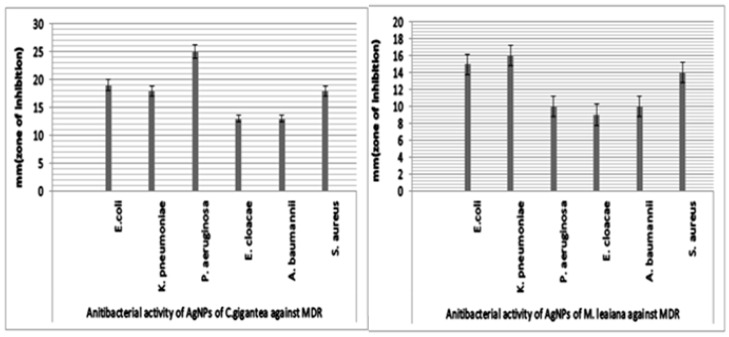
Antibacterial potential of silver NPs of *C. gigantea* (C) and *M. leaiana* (M) against MDR bacteria.

**Figure 11:**
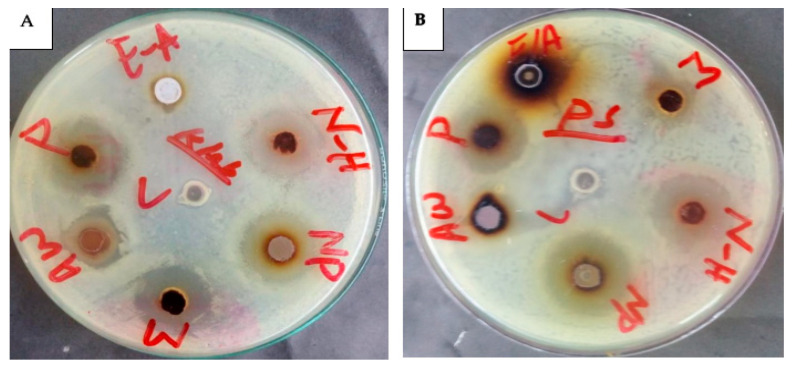
Antibacterial action of *C. gigantea* composites against *K. pneumoniae* (**A**) and *P. aeruginosa* (**B**) N-H = n-hexane (19 mm) and (22 mm); NP = nanoparticles (18 mm) and (25 mm); M = methanolic (23 mm) and (12 mm); Aw = aqueous water (16 mm) and (16 mm); P = pure water extract (20 mm) and (19 mm); E-A = ethyl acetate (0) and (0).

**Figure 12:**
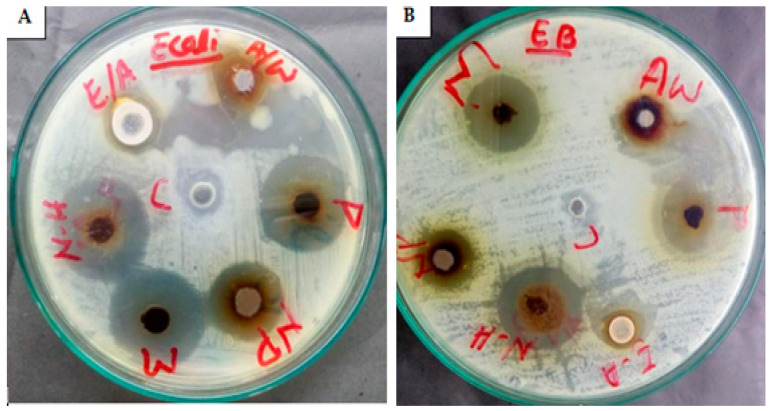
Antibacterial potential of *M. leaiana* composites against *E. coli* (**A**) and *E. cloacae* (**B**) N-H = n-hexane (19 mm) and (13 mm); NP = nanoparticles (15 mm) and (9 mm); M = methanolic (20 mm) and (20 mm); Aw = aqueous water (8 mm) and (14 mm); P = pure extract (19 mm) and (19 mm); E-A = ethyl acetate (10 mm) and (8 mm).

**Figure 13:**
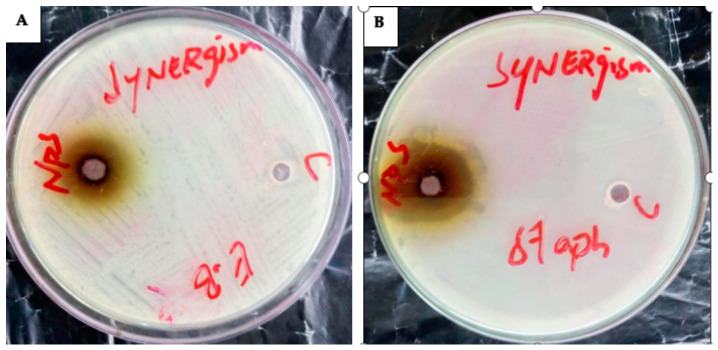
The synergetic effect of AgNPs against (**A**) = *E. cloacae* (E.B = 21 mm) and (**B**) = *S. aureus* (*staph.* = 29 mm).

The academic community has extensively proven that AgNPs could be the best alternative to antibiotics to control the infections triggered by MDR bacterial strains. AgNPs biosynthesized using mushroom extract were investigated for their antimicrobial effectiveness against various strains of pathogenic bacteria. The maximum ZOI of *C. gigantea* AgNPs was 25 mm against *P. aeruginosa*, and the minimum was 13 mm against *A. baumannii* and *E. cloacae* (Figure 10 and Figure 11). The highest ZOI of *M. leaiana* NPs was 16 mm against *K. pneumoniae* and the least was 9 mm against *E. cloacae* (see Table 1 and Table 2) (Figure 12 and Figure 13). Our study relates to a previous investigation, in which AgNP biosynthesized by *Malus domestica* had the highest activity against *K. pneumoniae* (23 mm), *P. aerogenosa* (22 mm) and *E. coli* (8 mm) [34]. *C. gigantea* and *M. leaiana* AgNPs had excellent antimicrobial effects against MDR pathogens but were less effective against Gram-positive compared to Gram-negative bacteria due to the bacterial cell wall composition, whereas lipopolysaccharides are covalently bonded lipids and polysaccharides. The weak positive charge on AgNPs is attracted to the negative charges on lipopolysaccharide. Gram-negative bacteria can be attracted toward negatively charged AgNPs via metal depletion [35]. In contrast, Gram-positive bacteria contain dense layers made up of peptidoglycan that are made of polysaccharide chains linked together by short peptides to create a rigid, three-dimensional structure. The cell wall’s rigidity and extensive cross-linking give the AgNPs fewer anchoring sites and make it more difficult for them to penetrate. The maximum antibacterial potential of *M. leaiana* AgNPs with the synergism of *C. gigantea* was noticed against *S. aureus* (29 mm), and the minimum was 20 mm against *P. aeruginosa*. The binding reaction between NPs and significant bioactive compounds like proteins, flavonoid, quinones, enzymes, phenols and peptides that interfere with bacterial rRNA may be the reason for the increase in synergistic effect. Because of their small size, it has been suggested that AgNPs made at 60 °C have a stronger antibacterial effect than those made at 25 °C [36]. Undoubtedly, the significant bactericidal activity is caused by the silver cations generated by AgNPs, which function as reservoirs for the silver’s bactericidal effect. The interaction with Ag cations significantly alters the pathogenic membrane structure, increases membrane penetrability and damages the bacterial cell wall by producing AgNP free radicals, eventually resulting in cell death [37,38].

### 2.6. Minimum Inhibitory Concentration

MIC was measured to conclude the effectiveness of both mushroom extracts and NPs.

**Figure 14:**
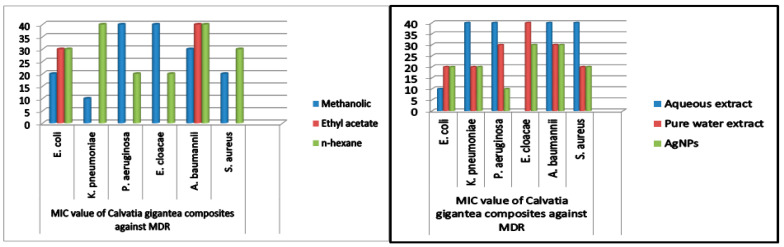
MIC values of *C. gigantea* against MDR bacteria.

**Figure 15:**
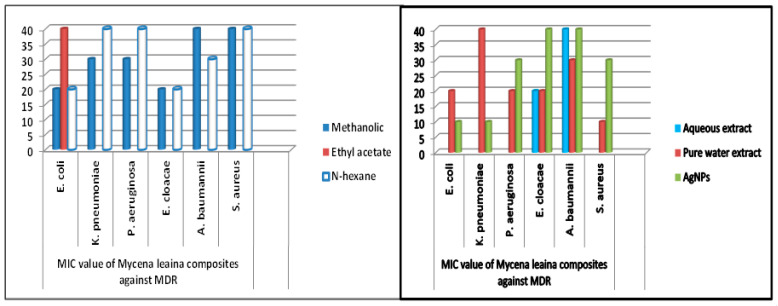
MIC values of *M. leaiana* against MDR pathogens.

The MIC values were typically low (10–40 mg/mL), demonstrating the extracts’ and NPs’ potency. The MIC of medicinal mushrooms is given in (Table 3 and Table 4). Methanolic extracts of *C. gigantea* and *M. leaiana* both had MIC values of 10 mg/mL against *K. pneumoniae* (Figure 14) and 20 mg against *E. coli* and *E. cloacae* (see Table 3 and Table 4 below) (Figure 15). Additionally, MIC values for n-hexane *C. gigantea* extracts against *P. aeruginosa and E. cloacae* were both 20 mg/mL and that *M. leaiana* against *E. coli* was 20 mg/mL (Figure 14). The MIC value of the ethyl-acetate extract of *C. gigantea* against *E. coli* was 30 mg/mL (Figure 14), and that *M. leaiana* against *E. coli* was 40 mg/mL (see Table 3 and Table 4 below) Figure 15). Contrary to expectations, *C. gigantea* AE’s MIC value against *E. coli* was 10 mg/mL (Figure 14), and that of *M. leaiana* against *E. cloacae* was 30 mg/mL. Pure water extract from *C. gigantea* had an MIC value of 20 mg against *K. pneumoniae*, *E. coli* and *S. aureus* (Figure 14), while that of *M. leaiana* had an MIC of 10 mg/mL against *S. aureus*. Because of the distinctions in the construction of the cell walls, Gram-positive bacteria show more vulnerability to a variety of substances as compared to Gram-negative bacteria. Several authors reported that phyto-constituents are more efficient than Gram-negative in comparison to Gram-positive pathogens [39,40]. Overall, it can be seen that there are more differences between the two mushroom extract results in terms of MIC values. NPs from *C. gigantea* against *P. aeruginosa* and *M. leaiana* for *K. pneumoniae* and *E. coli* both had 10 mg/mL MICs (Figure 14 and Figure 15). According to the results, *A. baumannii* has the maximum MIC values, indicating that it takes a larger number of NPs to cease its growth, compared to *E. coli* and *P. aeruginosa*, which exhibited lower MIC values, indicating that fewer NPs are needed to stop their bacterial growth. Due to the presence of impurities, antimicrobial bio-active substances obtained via green synthesis have a higher MIC potential than those derived from other synthetic sources [41,42]. On the determination of MIC values of mushroom composites and NPs, no work has been published in the literature.

### 2.7. Antibiotic Sensitivity

Antibiotic sensitivity, measured via a variety of antibiotics discs, along with the values for sensitivity (S), intermediate (I) and resistance (R), are provided in (Table 5).

Standard antibiotics affect cultures differently. Statistical investigation showed that mushroom extracts, excluding ethyl-acetate and AE, and the synergistic action of NPs were more effective against most bacterial strains than traditional antibiotics. *S. aureus* is more sensitive to ethyl-acetate than Gentamicin, Cefpirome and Chloramphenicol. *E. coli*, which is resistant to Chloramphenicol, Tazabactam, Cefepime, Ceftxime and Ceftazidime, was susceptible to mushrooms and NPs. All selected composites except ethyl-acetate inhibited *P. aeruginosa*, *E. cloacae*, *K. pneumoniae*, *A. baumannii*, *P. mirabilis* and *E. coli*.

## 3. Methodology

### 3.1. Collection of Mushroom

*C. gigentea* mushrooms were collected from their native habitat of Hunza, Gilgit-Baltistan, and *M. leaiana* mushrooms were collected from the local area, Khaki, Mansehra district. Mushroom specimens were washed and shade-dried for 18 days using a fan or gas heater, then ground to a powder and stored in small boxes with blotting sheets for further analysis [43].

### 3.2. Identification

#### 3.2.1. Morphological Study

An appropriate hand lens (10 × magnifications) was used to study the morphological characteristics at the location. Sporocarps and basidocarps from mushrooms were gathered and examined using an ocular micrometer in order to confirm mushroom species. Field notes were gathered on the essential macroscopic characteristics of the fresh specimens, such as color, structure and texture. Ocular micrometer was used for spore dimensions. Mushroom samples were stored by putting them in packets and tiny boxes containing blotting papers. The specimens were stored by drying them with a fan or a gas heater and preserving them in tagged packets or cartons with all of the information needed for later study [44].

#### 3.2.2. Anatomical Study

For anatomical study, tramal hyphae, pilies spore diameter, sporangium, cystidium and stupidities components were examined using a light microscope with a Lucida camera [45].

### 3.3. Laboratory Procedure for Identification of Mushroom

#### 3.3.1. DNA Isolation and Amplification

The extraction of DNA from dried fruit bodies of the mushroom samples was performed using the Extract N. Amp.TM Plant kit (Sigma-Aldrich Co., LLC, Saint Louis, MO, USA), slightly modifying the manufacturer’s methodology. The rDNA ITS region (Ribosomal Internal Transcribed Spacer section of the DNA) was amplified using species-specific forward and reverse primers ITS1F in combination with ITS4. PCR was carried out in 20 µL PCR tubes containing 10 µL 2× Ready mix (Sigma-Aldrich Co., LLC, Saint Louis, MO, USA), 8.3 µL water and 0.1 µL of each primer, as well as 1.5 µL of DNA extract. Next, 3 min of initial denaturation and 30 s of final denaturation at 95 °C were followed by 35 cycles of PCR at 53 °C for 35 s, initial extension for 1.35 + 5 s at 72 °C and final extension for 2 min at 72 °C [46].

#### 3.3.2. Sequencing and Blast Analysis

After sequencing, amplification and cloning, NCBI BLAST analysis was conducted. The sequences were aligned with retrieved gene bank sequences using MUSCLE and MAFFT online tools [47].

#### 3.3.3. Phylogenetic Analysis

Sequences alignment was performed using Bioedit 7.2 (Scotts Valley, CA, USA) since matching multiple indels was difficult, and all ambiguous indels were removed. For phylogenetic analysis, MEGA (6) program was used. MegAlign (DNA STAR) was used to examine rDNA-ITS region. From 1000 replicates, maximum likelihood (ML) and bootstrap consensus trees were produced using the Tamura-Nai model, with significant bootstrap values provided in the tree [48].

### 3.4. Extracts Preparation

Mushrooms were first washed properly using distilled water and kept in a shady area to dry for several weeks because mushrooms take a long time to dry. Then, the dried mushrooms were ground to powder form using an electric grinder and, for further experiments, stored in plastic zipper bags. Around 50 g of every composite was diluted in 250–400 mL of polarity-based solvents (n-hexane, ethyl acetate, methanol and distilled water). The solutions were incubated afterward in a shaker incubator for 72 h at 40 °C and 120 rpm. Nylon and Whatman filter paper was utilized to filter the extract mix. The obtained filtrate at 60 °C was rotary desolventized. A fractional crude extract was obtained after drying the semi-solid composite for 3 days in an oven at 60 °C. After removing the residue, final composites were refrigerated at 4 °C until antibacterial testing [49].

### 3.5. Pure Water Extract Preparation

The preparation of pure water composite was not based on the polarity. In a 1000 mL flask, 25 g fresh mushroom powder was added, followed by 300 mL distilled water and sealed with aluminum foil. The same procedure as described above was employed. Pure water extract was synthesized to compare bactericidal activity with polarity-based extract.

### 3.6. Synthesis of AgNPs

For AgNPs synthesis, silver nitrate (0.50961 g) was added to a 1000 mL beaker filled with distilled water (90 mL).

The silver nitrate molecular weight is 169.87 g/mol. Three-millimolar NPs were prepared according to following formula:

$$\frac{\mathrm{Molecular}\;\mathrm{weight}\;\mathrm{of}\;\mathrm{silver}\;\mathrm{nitrate}}{1000}\times 3\;\mathrm{mM}$$

Calculation = 169.87 gm/mol ÷ 1000 = 0.16987 g

= 0.16987 g × 3 mM = 0.50961

Then, a solution of 10 mL of mushroom aqueous composite was added to silver nitrate solution, and the synthesis medium was heated using hot plate magnetic stirrer for 48 h in order to ensure a complete reduction of metal ions [50]. When the solution color changed, AgNP synthesis was verified. The synthesis medium was centrifuged at 8000 rpm at 30 °C for 30 min following the completion of the 48 h. After centrifugation, the silver nanoparticles that formed a pellet at the bottom and the supernatant were discarded. Beakers were wrapped in aluminum foil and dried in an oven at 100 °C for 24 h. Finally, dried-out AgNPs were scraped from the beakers and kept in Eppendorf tubes at 4 °C [51].

### 3.7. Characterization of AgNPs

Different parameters were used for the characterization of produced AgNPs: UV-Vis spectroscopy, SEM, XRD, and FT-IR. The bioreduction of aqueous Ag ions was determined using a UV–visible spectroscopy analysis, carried out on a JASCO V-530, UV-Visible absorption spectrophotometer with a resolution of 2.0 nm between 200 and 600 nm and possessing a scanning speed of 300 nm/min. The processes of reactions between metal ions and mushroom extract were monitored by UV–Visible spectra of silver nanoparticles in aqueous solution. The X-ray diffraction technique was used to analyze the metallic nature of particles. After bioreduction, the silver nanoparticles solution thus obtained was purified via repeated centrifugation at 5000 rpm for 20 min, followed by redispersion of the pellet of silver nanoparticles into 10 mL of sterile deionized water. After freeze-drying of the purified silver particles, the structure and composition were analyzed using X-ray diffraction (XRD). The dried mixture of silver nanoparticles was collected for the determination of the formation of Ag nanoparticles via INEL X-ray diffractometer [52]. For FT-IR spectroscopy experiments, the residual solution after reaction was centrifuged at 10,000 rpm for 15 min, and the resulting suspension was repeated three times. After that, the purified suspension was washed with deionized water to obtain the pure form, i.e., free of proteins/enzymes which are not able to cap the silver nanoparticles. The sample was completely dried at 60 °C. Finally, the dried nanoparticles were analogued by FTIR (Nicolet iS50 spectrometer of resolution 4 cm^−1^). SEM was utilized to analyze particle shapes, surface morphology, sizes and size distributions of AgNPs [50].

### 3.8. Bacterial Strain

The bacterial strains *(E. coli*, *P. mirabilis*, *P. aeruginosa*, *K. pneumonia*, *E. cloacae* and *A. baumannii*) were maintained overnight in Luria Bertani liquid media, and 1 × 10^8^ cells mL^−1^ were maintained.

### 3.9. Antibacterial Test

The well diffusion technique was utilized to conduct antibacterial tests against MDR bacteria. The prepared media were mixed and autoclaved at 121 °C for 15 min. After cooling, media were added into Petri plates to solidify for 10 min. Then, seven wells were created in every plate, using a 6 mm cork borer, one for the negative control and six for the mushroom composites employed in the ratio of 1:60 dissolved in a solution containing 50% DMSO. Bacteria were inoculated on each Petri plate using a sterilized spreader or cotton swab. DMSO and double-distilled water solutions were used as controls [53].

### 3.10. Antibiotic Sensitivity

Antibiotics were used as a reference, including Gentamicin, Cefpirome, Chloramphenicole, Ceftxime, Tazobactam, Ceftazidime, Doxycycline and Cefepime.

### 3.11. Minimum Inhibitory Concentration

MIC of extracts and silver NPs were determined via broth dilution method. Antibacterial solutions were obtained by diluting mushroom extracts and AgNPs in DMSO and incubating them at 37 °C for 24 h. LB broth was added to each test tube. From each dilution, a concentration of 100 μL of extracts and AgNPs of mushrooms was serially diluted in LB broth. Then, one bacterial strain was inserted into all test tubes with four different extract dilutions. Test tubes were sealed using a sterile cotton stopper then kept incubated for 18 to 24 h at 37 °C. Spectrophotometry was used to calculate the rate of bacterial growth at 600 nm [OD] [54]. 

## 4. Conclusions

The seven bacterial strains employed in this study cause skin, wound, GI, urinary and respiratory infections. These MDR pathogenic bacteria were suppressed by a bioactive component in both higher fungi composites. The synergistic activity of mushroom composites opens up new treatment possibilities for infectious illnesses, allowing mushrooms to be employed as therapeutic agents, even when it is no longer effective itself. The findings clearly revealed that green-synthesized AgNPs may occasionally compete with commercial antibiotics for the treatment of pathogenic diseases. These environmentally friendly silver nanoparticles can be employed as an effective antibacterial agent against pathogenic microbes that are multi-drug resistant. However, additional research is needed, particularly on animal models, before they can be employed as antimicrobials. In the light of above conclusion, the following recommendation is presented for future studies: various chromatographic techniques, i.e., HPLC, TLC, GC-MS, etc., are strongly recommended for future work for the isolation of bioactive compounds which could lead to the discovery of innovative drugs.

## Figures and Tables

**Table 1:** *Calvatia gigantea*: Antibacterial activity (inhibition zones, mm) of the tested extracts.

Mushroom Composites (100 μL)	*E. coli*	*K. pneumoniae*	*P. aeruginosa*	*E. cloacae*	*A. baumannii*	*S. aureus*
Methanolic	22 ± 2.64	23 ± 1.73	12 ± 2.62	11 ± 1.62	18 ± 3.51	21 ± 2.64
Ethyl acetate	23 ± 1	R	R	R	16 ± 0.57	R
*n*-hexane	20 ± 1.72	19 ± 1.52	22 ± 3.51	18 ± 1.43	17 ± 2.53	16 ± 0.67
Aqueous extract	24 ± 3.05	16 ± 1.52	16 ± 2.08	R	15 ± 1.16	16 ± 2.51
Pure water extract	20 ± 3	20 ± 1.15	19 ± 2.28	17 ± 2.06	18 ± 1.76	20 ± 2.03
AgNPs	19 ± 2	18 ± 0.75	25 ± 1.52	13 ± 1.25	13 ± 0.56	18 ± 1.73

R: resistance.

**Table 2:** *Mycena leaiana:* Antibacterial activity (inhibition zones, mm) of the tested extracts.

Mushroom Composites (100 μL)	*E. coli*	*K. pneumoniae*	*P. aeruginosa*	*E. cloacae*	*A. baumannii*	*S. aureus*
Methanolic	20 ± 2.57	18 ± 1.16	17 ± 3.05	20 ± 1.25	15 ± 0.46	15 ± 0.57
Ethyl acetate	10 ± 1.87	4 ± 3	9 ± 0.62	8 ± 0.73	R	R
*n*-hexane	19 ± 2.62	10 ± 0.72	14 ± 0.57	17 ± 3.51	13 ± 1.35	16 ± 1.43
Aqueous extract	8 ± 2.08	5 ± 1.52	6 ± 1.15	14 ± 0.57	10 ± 0.53	R
Pure water extract	19 ± 2.57	16 ± 2.08	20 ± 0.17	19 ± 0.52	18 ± 0.53	22 ± 1.64
AgNPs	15 ± 0.57	16 ± 1.52	10 ± 1.43	9 ± 0.62	10 ± 1.36	14 ± 1.45
AgNPs synergy	22 ± 2.64	24 ± 2.08	20 ± 3.05	21 ± 3	23 ± 2.08	29 ± 2

R: resistance.

**Table 3:** MIC value of *Calvatia gigantea* composites against MDR bacteria.

Mushroom Composites Dilutions (100 μL)	MIC Values (mg mL^−1^)					
	*E. coli*	*K. pneumoniae*	*P. aeruginosa*	*E. cloacae*	*A. baumannii*	*S. aureus*
Methanolic	20 ± 0.179	10 ± 0.117	40 ± 0.127	40 ± 0.227	30 ± 0.139	20 ± 0.103
Ethyl acetate	30 ± 0.217	---	---	---	40 ± 1.13	---
*n*-hexane	30 ± 0.132	40 ± 1.46	20 ± 1.53	20 ± 0.971	40 ± 0.083	30 ± 0.471
Aqueous extract	10 ± 0.181	40 ± 0.134	40 ± 0.72	---	40 ± 0.107	40 ± 1.12
Pure water extract	20 ± 0.161	20 ± 0.503	30 ± 0.136	40 ± 0.127	30 ± 0.941	20 ± 1.08
AgNPs	20 ± 0.113	20 ± 0.145	10 ± 0.181	30 ± 0.929	30 ± 0.169	20 ± 0.209

**Table 4:** MIC values of *Mycena leaiana* composites against MDR bacteria.

Mushroom Composites Dilutions (100 μL)	MIC Values (mg mL^−1^)					
	*E. coli*	*K. pneumoniae*	*P. aeruginosa*	*E. cloacae*	*A. baumannii*	*S. aureus*
Methanolic	20 ± 0.119	30 ± 0.459	30 ± 0.138	20 ± 1.06	40 ± 0.171	40 ± 0.117
Ethyl acetate	40 ± 0.221	---	---	---	---	---
*n*-hexane	20 ± 0.147	40 ± 0.173	40 ± 0.093	20 ± 0.197	30 ± 0.203	40 ± 0.119
Aqueous extract	---	---	---	20 ± 0.186	40 ± 0.145	---
Pure water extract	20 ± 0.121	40 ± 0.136	20 ± 0.187	20 ± 0.127	30 ± 0.139	10 ± 0.163
AgNPs	10 ± 0.831	10 ± 0.651	30 ± 0.712	40 ± 0.143	40 ± 0.163	30 ± 0.209

**Table 5:** The area of antibiotics corresponding to CLSI guideline: resistance (R), sensitivity (S), intermediate (I).

Antibiotics	*E. coli*	*Staph*	*K. p*	*E. b*	*A. b*	*P. a*	*P. m*
Cefpirome (CPR)	R	R	R	S	S	I	I
Tazobactam (TZP)	R	S	R	S	S	I	S
Gentamicin (GEN)	S	I	R	S	S	I	R
Ceftazidime (CAZ)	R	R	R	S	R	I	R
Doxycycline (DO)	R	R	R	R	R	R	R
Cefepime (FEP)	R	R	R	R	R	R	R
Ceftxime (CFM)	R	R	R	R	R	R	R
Chloramphinicole (C)	R	I	S	R	I	R	S

## Data Availability

Not applicable.
