# Correction: Khan et al. Molecular Profiling, Characterization and Antimicrobial Efficacy of Silver Nanoparticles Synthesized from *Calvatia gigantea* and *Mycena leaiana* Against Multidrug-Resistant Pathogens. *Molecules* 2023, *28*, 6291

**DOI:** 10.3390/molecules29225225

**Published:** 2024-11-05

**Authors:** Sayab Khan, Muhammad Fiaz, Iqbal Ahmad Alvi, Muhammad Ikram, Humaira Yasmin, Junaid Ahmad, Amin Ullah, Zeeshan Niaz, Shubana Hayat, Ajaz Ahmad, Prashant Kaushik, Arshad Farid

**Affiliations:** 1 Department of Microbiology, Hazara University, Mansehra 21300, Pakistan; 2 Department of Botany, Hazara University, Mansehra 21300, Pakistan; 3 Jamil Ur Rehman Center for Genome Research, HEJ, ICCBS University of Karachi, Karachi City 75270, Pakistan; ikramkalyar@gmail.com; 4 Department of Infectious Disease, Faculty of Medicine, South Kensington Campus, Imperial College, London SW7 2BX, UK; 5 Department of Biosciences, COMSATS University, Islamabad 45550, Pakistan; 6 Department of Microbiology, Immunology, Infectious Diseases, Transplantation and Related Diseases, University of Rome Tor Vergata, 00133 Rome, Italy; 7 Department of Health and Biological Sciences, Abasyn University, Peshawar 25000, Pakistan; aminbiotech7@gmail.com; 8 Department of Clinical Pharmacy, College of Pharmacy, King Saud University, Riyadh 11451, Saudi Arabia; 9 Independent Researcher, 46022 Valencia, Spain; prashantumri@gmail.com; 10 Gomal Center of Biochemistry and Biotechnology, Gomal University, Dera Ismail Khan 29050, Pakistan

## Addition of Two Authors

“Iqbal Ahmad Alvi” and “Muhammad Ikram” were not included as authors in the original publication [1]. The corrected author contributions statement appears here:

**Author Contributions**: Conceptualization, S.K. and I.A.A.; methodology, S.K. and I.A.A.; validation, M.F., H.Y., A.U. and P.K.; formal analysis, M.I., H.Y. and A.F.; investigation, M.F., J.A. and Z.N.; resources, I.A.A., M.I. and Z.N.; data curation, A.U., S.H. and P.K.; writing—original draft preparation, S.K.; writing—review and editing, Z.N., A.A., P.K. and A.F.; visualization, A.U.; supervision, M.F., I.A.A. and Z.N.; funding acquisition, I.A.A. and A.A. All authors have read and agreed to the published version of the manuscript.

## Authors’ Information

This article has been republished with a minor update to the correspondence contact information and affiliation.

In the publication, there was an error regarding the affiliation for “Prashant Kaushik”. The updated affiliation should be as follows: “Independent Researcher, 46022 Valencia, Spain”.

The authors state that the scientific conclusions are unaffected. This correction was approved by the Academic Editor. The original publication has also been updated.
